# A Microfluidic Chip Architecture Enabling a Hypoxic Microenvironment and Nitric Oxide Delivery in Cell Culture

**DOI:** 10.3390/mi11110979

**Published:** 2020-10-30

**Authors:** Samineh Barmaki, Daniela Obermaier, Esko Kankuri, Jyrki Vuola, Sami Franssila, Ville Jokinen

**Affiliations:** 1 Department of Pharmacology, Faculty of Medicine, University of Helsinki, 00290 Helsinki, Finland; esko.kankuri@helsinki.fi; 2 PreSens Precision Sensing GmbH, 93053 Regensburg, Germany; Daniela.Obermaier@presens.de; 3 Helsinki Burn Centre, Jorvi Hospital, Helsinki University Hospital and University of Helsinki, 00260 Helsinki, Finland; jyrki.vuola@helsinki.fi; 4 Department of Chemistry and Materials Science, School of Chemical Engineering, Aalto University, 00076 Espoo, Finland; sami.franssila@aalto.fi (S.F.); ville.p.jokinen@aalto.fi (V.J.)

**Keywords:** hypoxia, nitric oxide, microenvironment, cell culture, microfluidic chip, oxygen depletion, sodium nitroprusside, gasotransmitter

## Abstract

A hypoxic (low oxygen level) microenvironment and nitric oxide paracrine signaling play important roles in the control of both biological and pathological cell responses. In this study, we present a microfluidic chip architecture for nitric oxide delivery under a hypoxic microenvironment in human embryonic kidney cells (HEK-293). The chip utilizes two separate, but interdigitated microfluidic channels. The hypoxic microenvironment was created by sodium sulfite as the oxygen scavenger in one of the channels. The nitric oxide microenvironment was created by sodium nitroprusside as the light-activated nitric oxide donor in the other channel. The solutions are separated from the cell culture by a 30 µm thick gas-permeable, but liquid-impermeable polydimethylsiloxane membrane. We show that the architecture is preliminarily feasible to define the gaseous microenvironment of a cell culture in the 100 µm and 1 mm length scales.

## 1. Introduction

The cellular microenvironment is the small-scale environment in the immediate vicinity of cells. Gaseous transmitters, such as oxygen (O_2_), hydrogen sulfide (H_2_S), carbon monoxide (CO), and nitric oxide (NO), have several major physiological and pathological roles in the body [1,2,3]. In the cells’ normal tissue microenvironment, the oxygen content ranges between 2 and 6% [4]. Another important biological regulator is nitric oxide, a fundamental component in many fields of physiology and medicine. The main biological functions for nitric oxide are smooth muscle relaxation and inhibition of platelet aggregation and adhesion [5,6,7,8].

Control of gaseous microenvironments is a challenging task because of the high diffusivity of gases. Traditional hypoxia chambers cannot achieve rapid gas level modulation because of the large volume of gas to be exchanged [9,10]. On the other hand, direct addition of donor or scavenger chemicals into cell growth medium alters the composition of the medium, leading to potential toxic effects or other artifacts. Furthermore, both methods are only suitable for changing the gaseous environment of the entire cell culture without the possibility to create spatially patterned microenvironments.

Recently, microfluidics has been used for manipulating and controlling cellular gaseous microenvironments for in vitro and in vivo studies [11,12,13,14,15]. Microfluidics is a discipline that uses micro- or nanoscale channels for mixing, delivering, and sorting of fluids. Microfluidics has been utilized to create gradients of oxygen or other gases [14,16,17,18,19,20,21]. Oppegard et al. [22] utilized a gas permeable polydimethylsiloxane (PDMS) microfluidic insert for cell culture well plates to control the oxygen concentration inside the well. By utilizing two interdigitated meanders (two parallel channels), they were able to create mm scale areas of 21%, 10%, and 0% oxygen gradients inside the well. Cell proliferation resulted in 10% oxygen, but cell death was observed in 0% oxygen after five days. Another study showed that oxygen gradients could be generated for cell culture by controlling chemical reactions in a microfluidic channel to scavenge oxygen in a neighboring cell culture channel. The microchip device consisted of a central cell culture that is flanked on either side with a chemical reaction channel. The oxygen gradient was formed in the central channel. The oxygen was generated by the reaction of hydrogen peroxide with sodium hypochlorite (H_2_O_2_ + NaOCl) and the oxygen scavenging reaction by pyrogallol + NaOH. Each chemical entered the chip through a dedicated channel [19].

Funamanto et al. [23] reported a PDMS microfluidic device for the cell culture in a 3D hypoxic environment. The device created a uniform low oxygen gradient and was used for imaging of human breast cancer cell migration in 3D format. First, the gas mixture of 21% oxygen, 5% carbon dioxide, and 74% nitrogen was supplied through gas channels to create a normoxic condition, then hypoxia was generated by switching the gas mixture from 21% oxygen gas mixture to 0% oxygen gas mixture through gas channels for >8 h to monitor cell migration. Skolimowski et al. [24] presented the generation of oxygen gradient in the microchip setup with a gas permeable membrane. Oxygen scavenging liquid (10% sodium sulfite solution with 0.1 mM CoSO_4_ as catalyst) was pumped in a microfluidic channel and generated the desired oxygen, which was used for mimicking physiologically and biologically relevant in vivo environments [25]. Alder et al. [14] utilized a microfluidic device made of PDMS with two layers of microchannels and a computer controlled multi-channel gas mixer. The device had nine gas inlets, fed by O_2_–N_2_ mixture. The gas mixture was computer-controlled and is set individually in each of its channels, making it possible to generate [O_2_] gradients with linear, exponential, and nonmonotonic profiles in a single device. This system can be used to study migration of cells under oxygen gradients.

In addition to hypoxia, microfluidics has been used to control the concentrations of other gases, such as nitric oxide (NO) and carbon dioxide (CO_2_). Chen et al. [15] generated stable nitric oxide (NO) gradients in the cells by utilizing chemical reactions in PDMS, a gas permeable microfluidic device. NO was produced by the chemical reaction between nitric acid and silver. Cells were cultured inside the device and the fluorescence dye, DAF-FM (Diaminofluorescein-FM diacetate), was used for observing NO concentration in the cells. The results suggested that the device could be used to study cell behaviors under different levels of NO concentrations without using gas cylinders. The controlled carbon dioxide by a PDMS microchip was demonstrated by Forry et al. [26]. The desired levels of carbon dioxide partial pressure (PCO_2_) for mammalian cells were generated in minutes and retained for days.

In our previous work [27], we showed a configuration where hypoxia was induced on HEK-293 cells cultured in a well that was separated from an underlying oxygen scavenging channel network by a thin PDMS membrane. Here, we provide a first insight into a microfluidic ship architecture for simultaneous patterning of oxygen and nitric oxide microenvironments in cell culture. We utilize two separate, but interdigitated channels to deliver the oxygen scavenger and nitric oxide donor solutions to the desired locations.

## 2. Materials and Methods

### 2.1. Chip Fabrication

The PDMS chips were fabricated by replication moulding from SU-8 masters. The steps to make the master were as follows: spin coating SU-8 50 on top of silicon wafers (4000 rpm, 30 s), soft baking (95 °C, 20 min), exposure for 12 s, post exposure bake (95 °C, 10 min), and development. The thickness of the masters, and thus the height of the microchannels used, was 40 µm.

**Figure 1:**
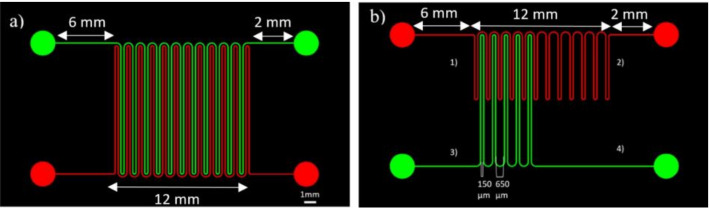
Microfluidic chip design. (**a**) The main chip type containing two separate, but interdigitated channels. The channels are 100 µm wide and the gap between two channels is 150 µm. The channel height is 40 µm. (**b**) A chip variant used for mm scale patterning of four different microenvironments: (1) hypoxia and nitric oxide, (2) only hypoxia, (3) only nitric oxide, and (4) neither treatment. The channels have two gap dimensions, 150 µm and 650 µm.

The PDMS chips were made from PDMS (Sylgard 184, Merck KGaA, Darmstadt, Germany) mixed with a 10:1 monomer to crosslinking agent ratio. PDMS was poured on top of the master, degassed in vacuum, and cured in an oven at 70 °C for 2 h. The geometry of the main chip utilized is shown in Figure 1a and the alternative chip used for demonstrating spatial patterning of gas microenvironments in the cm scale is shown in Figure 1b. The channel width is 100 µm, the spacing between channels is 150 µm, and the length of the channel is 20 mm.

A 30 µm thin PDMS membrane was made from a 10:1 ratio Sylgard 184 by spin-coating. The degassed mixture was spin-coated on a fluoropolymer coated silicon wafer (2000 rpm 2 min), after which the membrane was cured in an oven at 70 °C for 2 h. To assemble the chip, the microfluidic chip was plasma bonded to the thin membrane. This was done by exposing both to oxygen plasma (60 W, 500 mL/min O_2_, 1 min, PVA Tepla plasma reactor, Wettenberg, Germany). Immediately after the plasma treatment, the chips were placed on top of the membrane and the bonding was finished in an oven at 70 °C for 10 min. After cooling to room temperature, the bonded chips were peeled off the fluoropolymer coated silicon wafer. The cell reservoirs placed on top of the membrane were carved from a ≈1 cm thick PDMS block with a knife.

### 2.2. Oxygen Measurements Using Oxygen Sensing Foil

The VisiSens system (PreSens Precision Sensing GmbH, Regensburg, Germany) was used for imaging of oxygen levels. This system consists of a detection camera and O_2_-sensitive sensor membranes. VisiSens AnalytiCal 1 software (VA1.12, PreSens GmbH, Regensburg, Germany) was used for analysis of the results. For experiments on microfluidic chips, the sensor membranes were coated with PDMS and then integrated to the chip. The method for preparation of oxygen scavenger water and analyzing oxygen signals in a microchip on oxygen sensor foil were explained in our previous publication [27]. To calibrate the sensor, the PDMS membrane was coated on oxygen sensor foil and calibrated with oxygen-depleted water (835 mM Na_2_SO_3_) as 0 level (marked as black in the images) and oxygen saturated water as 100 level (marked as white in the images). The size of the droplets was 1–10 µL.

### 2.3. Preparation of Microfluidic Chips Setup

Before experiments, the microfluidic chips and cell reservoir were washed with soap and rinsed with deionized water and 70% ethanol. The chips were dried with pressurized air. Then, a piece of scotch tape was used to clean away any remaining dust or particles. For testing the functionality of microfluidics before cell culture experiments, water was pumped with a microfluidic syringe pump (NE-4000, New Era Pump Systems Inc, Farmingdale, NY, USA) with a flowrate of 0.1 mL/h or 1.6 µL/min into the microfluidic channels. If any leaks were detected during this test, the chip was discarded.

### 2.4. Cell Culture Experiments on Microchips

HEK-293 cells (ATCC CRL-1573, Manassas, V) from passage 6–20 were used for the experiments. Cells were cultured in Dulbecco’s Modified Eagle Medium (DMEM, Gibco, Thermo Fisher Scientific Inc, Waltham, MA, USA) with 10% fetal bovine serum (FBS, Gibco) and antibiotics (penicillin, streptomycin). The microfluidic chips were prepared for cell culture experiments in the same way as in our previous publication [27].

Bovine fibronectin (F1141, Sigma Aldrich, Germany) with a final concentration of 33.3 µg/mL was used for coating of the reservoir on top of the microfluidic chip. The coating was made by incubating the fibronectin solution for 2 h before the experiment. Afterwards, fibronectin was removed, and the cell reservoir was washed three times with PBS. HEK 293 cells (1 × 10^6^ cells/mL) with culture medium were added to the reservoir on the microfluidic chip and grown for 24 h before starting the experiment.

### 2.5. Hypoxia Response

Based on our previous publication [27], we used sodium sulphite to generate hypoxia (shown in Chemical Formula (1).

(1) $$2Na_{2}SO_{3}+O_{2}\to 2Na_{2}SO_{4}$$

To visualize hypoxia in cells, Image-iT^®^ Hypoxia reagent (Thermo Fisher Scientific, Waltham, MA, USA) was used with a final concentration of 5 µM. This reagent starts display fluorescence while the oxygen levels decrease in the cells from 5% [27,28]. HEK cells were cultured in the reservoir on the microfluidic chip and, after 24 h incubation, the medium was replaced with 800 µL of fresh culture media containing 5 µM Image-iT^®^ Hypoxia reagent. The microfluidic chip setup was incubated under the cell culture chamber of Nikon Eclipse Ti-EN- STORM microscope (Nikon, Nikon Instruments Europe BV, Amsterdam, The Netherlands) with 5% CO_2_ at 100× magnification. The oxygen depleted water started to pump with a flowrate of 0.1 mL/h for 7 h. During pumping, images were recorded from cells with 15 min intervals.

To evaluate the hypoxia response from one or two channels, data were collected from two individual experiments (*n* = 2).

### 2.6. Measurements of Nitrite on Microchip

There are several potential nitric oxide molecule donors, such as SIN-1 and sodium nitroprusside (SNP), which degrade under light and release nitric oxide [29].

Sodium nitroprusside dehydrate ${\left[\mathrm{Fe}\left(\mathrm{NO}\right){\left(CN\right)}_{5}\right]}^{2-}\;$ is one of the nitric oxide molecule donors used to release nitric oxide under light (Chemical Formula 2).

(2) $${\left[\mathrm{Fe}\left(\mathrm{NO}\right){\left(CN\right)}_{5}\right]}^{2-}\;\;\overset{\;\;\;\;\;\;e^{-}\;\;\;\;\;\;\;}{\to }{\left[\mathrm{Fe}\;\left(\mathrm{NO}\right){\left(CN\right)}_{5}\right]}^{3-}\;\overset{\;\;\;\;\;\;\;\;}{\to }\;\;{\left[\mathrm{Fe}\;{\left(\mathrm{CN}\right)}_{4}\right]}^{2-}\;+\;\mathrm{NO}+\;CN^{-}\;\;\;\;\;$$

Griess reaction is the most widely used method to detect nitrite and nitrate in physiological fluids. In this reaction, nitrite was chemically transformed into coloured diazo compound by reaction with sulphanilamide and N-(1-naphtyl) ethylenediamine. The reaction has two steps: the reduction of nitrate to nitrite and the derivatization and spectrophotometric detection of nitrite [30].

For generation of nitrite and nitric oxide, sodium nitroprusside (SNP) (567538 Sigma-Aldrich Merck KGaA, Darmstadt, Germany) with a final concentration of 20 mM was pumped with a syringe pump in microfluidic channels with a flowrate of 0.1 mL/h.

The sensitivity of sodium nitroprusside (SNP) to light (600 lumens-3 WATS LED flashlight zoom able modes aluminium lighting CREE Q5 ZOOM-JR international) degrades under light and releases nitric oxide. The maximum efficacy of SNP to produce NO under light was observed after 3 h. Because the half-life of nitric oxide is less than 30 s, nitrite measurements were used as an indicator of nitric oxide. 

For nitrite measurements, the culture medium was added to the reservoir on top of the microchip. SNP was pumped into the microfluidic channels, light was flashed precisely on top of reservoir, and the syringe pumps and tubing were protected from light to prevent the degradation of SNP during the experiment. The reservoir on top of the chip was filled with 800 µL of media. Nitric oxide was released from SNP degradation in the channels and crossed from the gas permeable thin PDMS layer to the reservoir.

**Figure 2:**
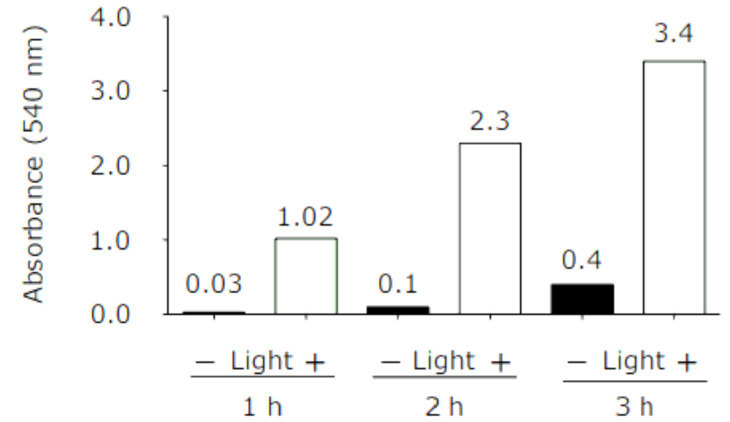
Light dependency of sodium nitroprusside (SNP) to degrade and release nitric oxide (NO). NO increased by time and the maximum release of NO in our study was observed from SNP under light after 3 h.

The light dependency of NO generation and NO retrieval from the cell culture compartment of the microfluidic chip is shown in Figure 2. The amount of NO generated in the cell culture well compartment of the microchip setup was quantified as nitrite concentration in the incubation medium. The light-sensitive NO-donor, SNP (20 mM) solution, was pumped into both channels of the chip for three hours, and nitrite concentrations were determined hourly with or without light activation.

In every hour, the media was mixed gently in the reservoir and 50 µL of media was taken from the reservoir, while Griess reagents (ab234044, Abcam, UK) with a total volume of 50 µL were added to the media and the nitrite absorbance value was measured by a spectrometer (540 nm wavelength). The minimum nitrite absorbance value was observed after 1 h and the maximum value was observed after 3 h of pumping of SNP in microfluidic channels.

**Figure 3:**
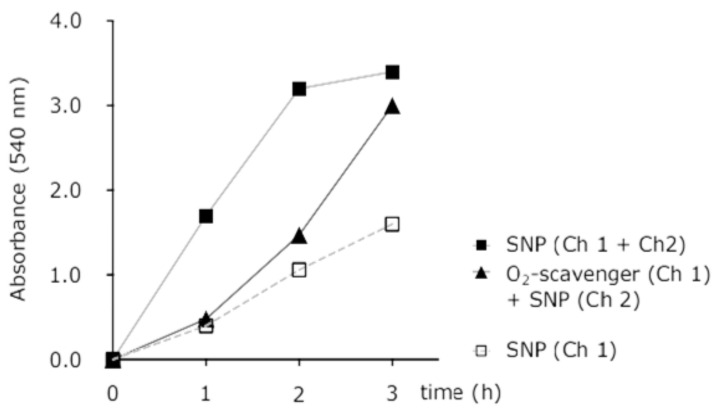
Time dependence of light-activated nitrite release from SNP in the microfluidic channels with or without concurrent hypoxia.

Figure 3 shows the time dependency of nitrite release from SNP in the microchip with and without concurrent hypoxia. This figure represents the difference in nitrite response while the chip is pumped with; SNP in two channels, SNP in one channel, SNP in one channel and oxygen depleted water in other channel. This test was done before cell experiments to observe and analyse the difference in nitrite response (as NO indicator) while SNP is pumped in one channel and two channels, as well as by pumping SNP and oxygen scavenger solution simultaneously through dedicated channels.

### 2.7. Nitric Oxide Response in Cells

DAF-2 DA, fluorescent nitric oxide probe (ab145283, Abcam, UK) with a final concentration of 10 µM was used to evaluate nitric oxide in the cells. This probe begins to display fluorescence when the nitric oxide generates in the cells. After 24 h of cell growth in the reservoir on the microchip, DAF-2DA was mixed with 800 µL fresh culture media and incubated in the dark for 20 min before the experiment. In the same manner as in hypoxia experiments, Nikon Eclipse Ti-EN- STORM microscope used for nitric oxide imaging and light was turned off before each 15 min interval. To evaluate the nitric oxide response from one or two channels, data were collected from two individual experiments (*n* = 2).

### 2.8. Simultaneous Recording of NO and Hypoxia Signals

The microchip setup and HEK cells were prepared in the same manner as in hypoxia and nitric oxide experiments. Cells reached ~80% confluence after 24 h. DAF-2 DA probe with a final concentration of 10 µM was added to the cells with 800 µL of fresh media and incubated for 20 min. Media was refreshed in the cells with Image-iT^®^ Hypoxia reagent with a total concentration of 5 µM. To recognize the hypoxia in the cells, first, oxygen-depleted water was pumped in one channel and water in the other channel. After visualizing the hypoxia from cells, SNP started to pump in the channel, which was pumped with water. The pumping flowrate of the syringe pump in all experiments was 0.1 mL/h. The graph of simultaneous response of hypoxia and nitric oxide was represented from an average of three individual experiments (*n* = 3) and the error bars were measured from standard deviation. Microscope experiments for observing nitric oxide in the cells (Figure 5c,d and Figure 6) while nitric oxide started to pump in the other channel] were done after exposure to light source.

### 2.9. Image Analysis

Images were analysed by Fiji ImageJ software version 1.8.0-172/ 1.53 c [31,32]. For analysing the hypoxia (red) and nitric oxide (green) signals in the cells, the composite colour mode in Fiji was chosen. The histogram of red, green, and phase contrast was edited to remove the image background, then the mean intensity versus time was analysed in each channel. The spatial variation of hypoxia and nitric oxide cell responses on the microchip was analysed by sectioning the image to 100 µm wide vertical stripes. These sections were defined as ROIs (regions of interest), then the mean intensity and standard deviation of the ROIs were analysed by Fiji.

## 3. Results and Discussion

### 3.1. Simultaneous Hypoxia and Nitric Oxide Response in Cells

**Figure 4:**
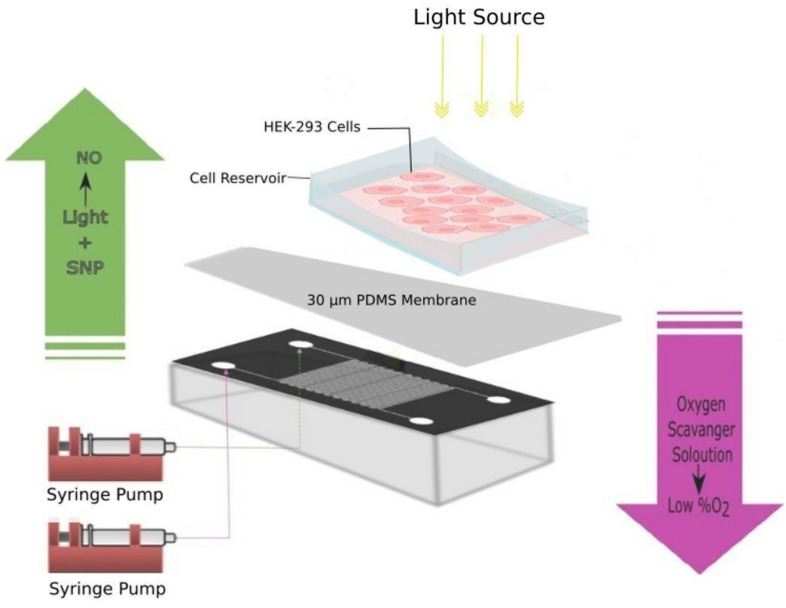
Microfluidic chip setup for cell experiments. PDMS, polydimethylsiloxane.

**Figure 5:**
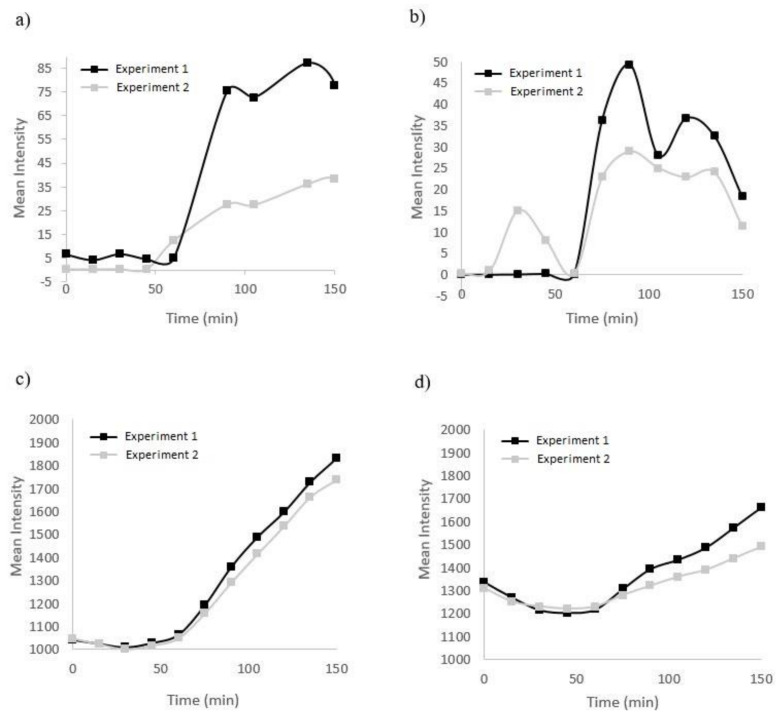
Fluorescence signal from hypoxia and nitric oxide in the cells. (**a**) Hypoxia in one channel; (**b**) hypoxia in two channels; (**c**) nitric oxide in one channel; (**d**) nitric oxide in two channels. The black and grey datasets are two independent experiments.

The microfluidic setup is shown in Figure 4. The microfluidic chip consists of two interdigitated meandering channels that are 100 µm wide, 40 µm in depth, and 20 mm in length. The channels were separated from a cell reservoir located on top of the channels by a 30 µm thick PDMS membrane. First, we characterized the ability of the chip to separately create either a hypoxic or nitric oxide response in cells using either both channels or just one channel, and the results are shown in Figure 5a–d.

Figure 5a shows that the oxygen levels started to decrease in the cells by pumping oxygen scavenger solution inside of one channel (flowrate: 0.1 mL/h). Intracellular hypoxia levels started to increase after 100 min and stabilized after 2 h in the cells. In Figure 5b, oxygen scavenger solution was pumped into both channels and the hypoxia in the cells started to increase after 1 h. The nitric oxide response is shown in Figure 5c,d. In Figure 5c, DAF-2 signal, as an indicator of nitric oxide in the cells, started to increase by pumping SNP into one channel, and Figure 5d indicates the nitric oxide signal in the cells after 100 min of pumping SNP in both channels.

**Figure 6:**
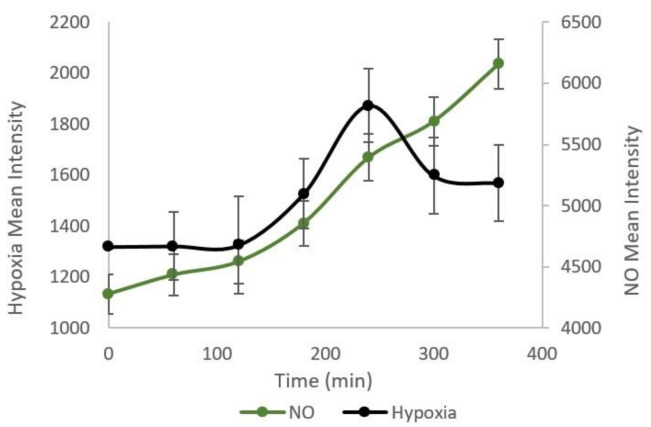
Simultaneous hypoxia and nitric oxide response from the cell culture. Simultaneous fluorescence signal from hypoxia and nitric oxide increased in the cells after 3 h of pumping O_2_-depleted H_2_O and SNP in meanders. The data were gathered from three independent experiments (n = 3) and error bars were measured from standard deviation.

Next, we demonstrated dual cellular gaseous microenvironments (NO and hypoxia) in the cells on chip by utilizing one meander for each gas. The results are shown in Figure 6. The cells started to show hypoxia and nitric oxide response nearly simultaneously around 100 min after the pumping of oxygen scavenger solution and SNP. Both signals remained elevated for 5 h until the end of the experiments. This result shows that the two chemicals in adjacent channels do not interfere with each other and that both treatments can be applied at the same time.

**Figure 7:**
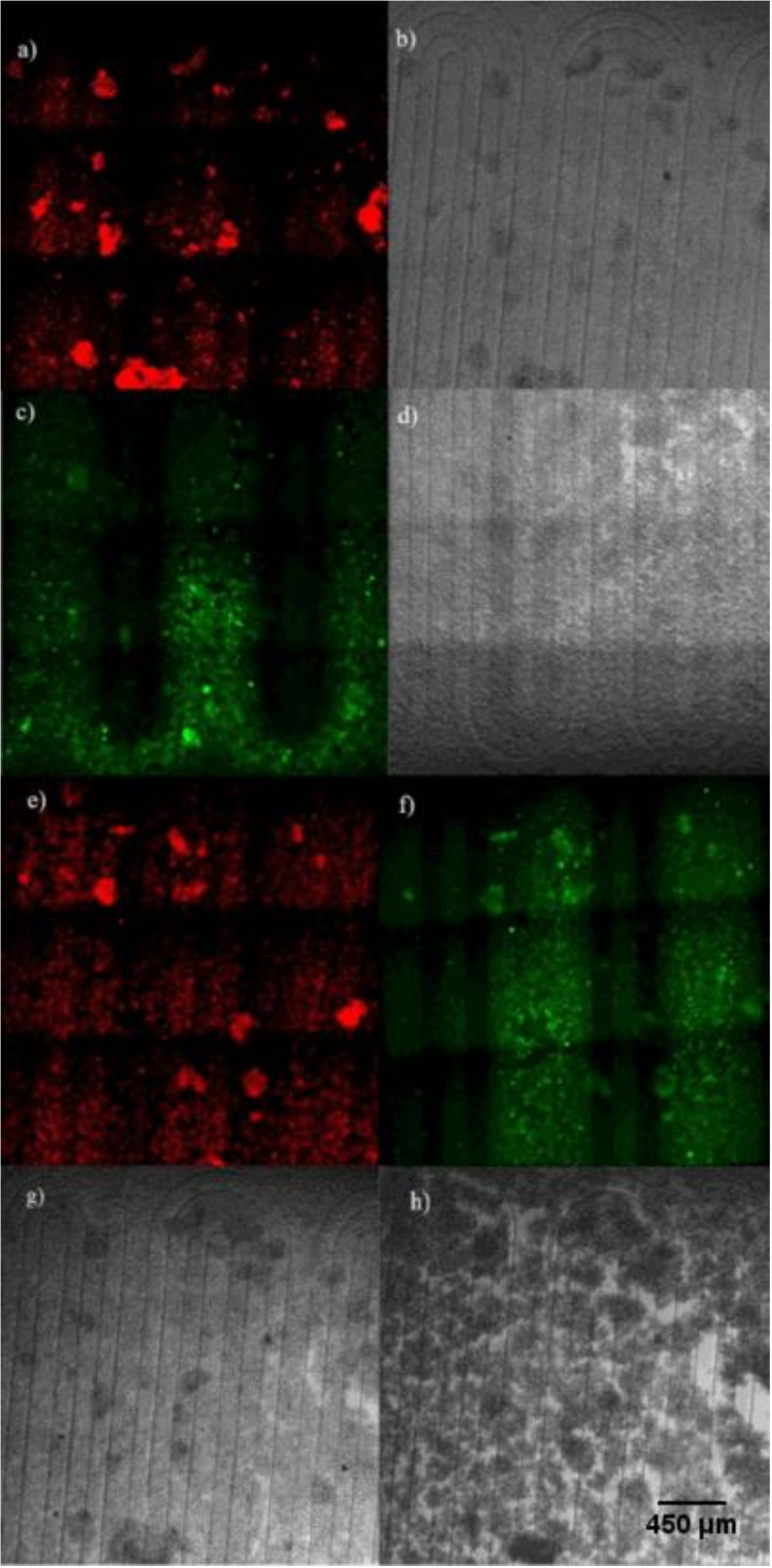
Combined patterning of hypoxia and nitric oxide microenvironment. (**a**) Hypoxia signal in the hypoxia only experiment. (**b**) Optical image of the cells in the hypoxia only experiment. (**c**) Nitric oxide signal in the nitric oxide only experiment. (**d**) Optical image of the cells in the nitric oxide only experiment. (**e**) Hypoxia signal in the combined experiment. (**f**) Nitric oxide signal in the combined experiment. (**g**) Phase contrast image of the cells before the simultaneous hypoxia and nitric oxide experiment. (**h**) Phase contrast image of the cells after the simultaneous hypoxia and nitric oxide experiment (image magnification: 100×).

Figure 7a,b shows the cellular hypoxia response by pumping oxygen scavenger solution in one channel. Figure 7c,d shows nitric oxide response in the cells by pumping SNP in one channel. Figure 7e,f shows the dual gaseous cells’ responses of hypoxia and nitric oxide on the microchip by simultaneously pumping the oxygen scavenger solution in one channel and SNP in the other channel. The morphology of the cells before and after starting the experiments is shown in Figure 7g,h.

### 3.2. Microenvironment Patterning

**Figure 8:**
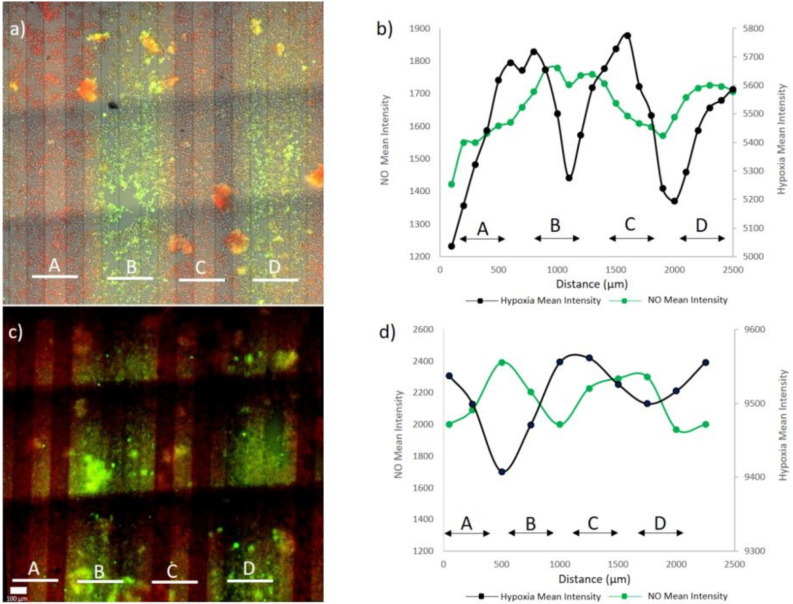
Simultaneous response to hypoxia and nitric oxide on a microchip. (**a**,**c**) Hypoxia and nitric oxide signals from cells simultaneously. (**b**,**d**) Spatial variation of hypoxia and nitric oxide cell responses on the microchip. The result shown in (**a**) is analyzed in (**b**) and the result shown in (**c**) is analyzed in (**d**).

The combined oxygen and nitric oxide microenvironments in Figure 8 show clear spatial variability in the signal intensities depending on the distance of the cells from the corresponding microfluidic channel. We analyzed the distance the treatments diffuse from the channels by analyzing these images. The hypoxia response (Figure 8a) is observed in cells throughout the cell culture, but the response was stronger in cells that were located close to the channels with the oxygen scavenger. In the case of nitric oxide (Figure 8c), most of the signals came from cells very close to the channel with the nitric oxide donor. We observe that the 150 µm gap between two adjacent nitric oxide donor channels was not visible, so the nitric oxide microenvironment spreads at least 75 µm from the channels. Neither treatment fully covered the 650 µm gap that forms on the areas where the channels for the other reagent are located (see Figure 1b for explanation of the 150 µm and 650 µm gaps). However, it seems that the hypoxia signal spreads slightly further from the channels, because the 650 µm gaps are better covered (Figure 8a,e) compared with the nitric oxide signal (Figure 8c,f). The diffusion coefficients of O_2_ and NO in water are very similar, around 2 × 10^−5^ cm^2^/s [33], and the diffusion coefficient of O_2_ in PDMS, that is, 3.2 × 10^−5^ cm^2^/s [34], is somewhat higher, but of the same magnitude that has been reported for NO in silicone rubber, that is, 1.6 × 10^−5^ cm^2^/s [35].

Comparing Figure 7a,c to Figure 7e,f shows that there is no major difference in the signals when obtained from separate experiments or from an experiment where both reagents are utilized simultaneously, showing that both treatments can be applied independently without interfering with each other. The monolayer structure of the cells in the beginning of the experiment (Figure 7g) starts to change after pumping oxygen scavenger solution and SNP. Oxygen scavenger solution reduces oxygen levels in the cells. NO production in the cells interferes with hypoxia signaling and inhibits the expression of hypoxia-inducible factor 1α (HIF-1 α) by pumping SNP and, over time, it causes the cells to clump up to some extent (Figure 7h). To quantify the spatial distribution of the microenvironment, we analyzed the average intensity as a function of the spatial coordinate in a direction perpendicular to the channels. The results are shown in Figure 8. Supplementary Video S1 shows the case analyzed in Figure 8a,b. The analysis shows that the maximum hypoxia signal was obtained from the cells growing on top of the oxygen scavenger channels and the maximum nitric oxide signals are obtained from cells growing on top of the nitric oxide donor channels. The lowest signal for both treatments was observed on the areas furthest away from the channels providing the respective treatment (in the middle of the channels for the other treatment). Between these points, there are cells exposed to various intensities and combinations of both treatments.

**Figure 9:**
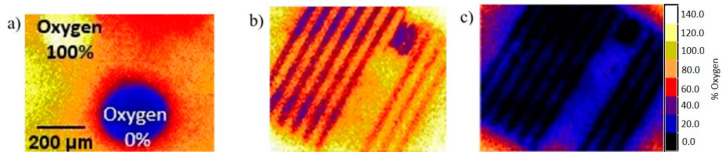
Oxygen measurements of microchip on oxygen sensor foil. (**a**) Calibration of oxygen on oxygen sensor foil with standards: oxygen-depleted water (0% oxygen: black colour) and oxygen saturated water (100% oxygen: white colour). (**b**) Oxygen signal from pumping one channel with oxygen depleted water. (**c**) Oxygen signal from pumping two channels with oxygen depleted water.

The hypoxia micro-pattern generated by the chip was further characterized by another setup by bonding the chip directly on top of an oxygen sensor foil, Figure 9. The calibration droplets with 100% and 0% oxygen levels are shown in Figure 9a. First, oxygen scavenger solution was pumped in one channel (Figure 9b), and then simultaneously in two channels (Figure 9c) with a flowrate of 1.6 µL/min. By pumping oxygen scavenger solution in one channel, oxygen signals (%Air. Sat.) started to decrease from 60 to 10. Then, by pumping oxygen scavenger solution simultaneously in two channels, oxygen signals decreased from 100 to 0. It should be noted that this experiment did not contain any cells and the results are not directly comparable to the intracellular results in Figure 5, Figure 6, Figure 7 and Figure 8. However, it still shows a similar result in that, with the dimensions chosen, the hypoxia signal can diffuse to the whole small 150 µm gap, but not the larger 650 µm gap. Overall, the results shown in Figure 7, Figure 8 and Figure 9 demonstrate simultaneous micro patterning of both the hypoxia and the nitric oxide microenvironment in the ≈100 µm size scale defined by the geometry of the channels and the gaps between the channels.

### 3.3. Microenvironment Patterning over Millimetre Scale

**Figure 10:**
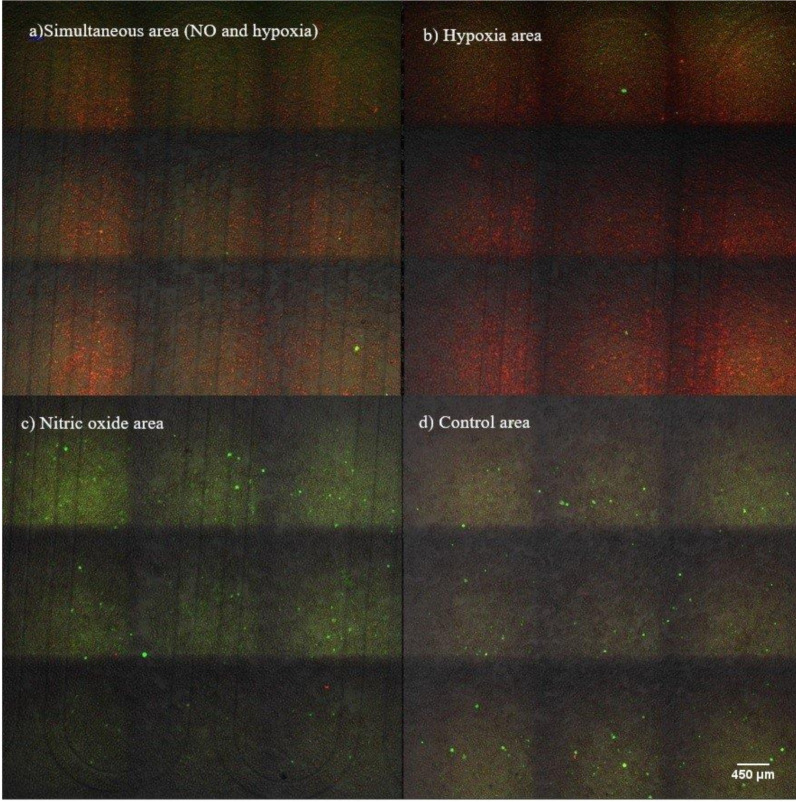
Fluorescence signals from the four different microenvironments achieved by the second microchip design. (**a**) Simultaneous response to nitric oxide and hypoxia. (**b**) Only hypoxia cell response. (**c**) Only nitric oxide cell response. (**d**) Signal from control area without treatments (image magnification: 100×).

Patterning of gaseous microenvironments on a more macroscopic mm to cm scale is achieved by tailoring the channel architecture. We demonstrate this by a second chip design, shown in Figure 1b, which is designed to divide the 1 cm × 1 cm cell culture into four roughly equally sized areas. The chip has four distinct 5 mm × 5 mm areas with both hypoxia and nitric oxide channels, or only one of the channels or no channels at all. These four cases represent all 2 × 2 combinations of hypoxia on/off, nitric oxide on/off. The results of this experiment are shown in Figure 10. Cells on the area exposed to both hypoxia and nitric oxide show a strong signal for both (Figure 10a), whereas cells exposed only to hypoxia or nitric oxide show dominantly only the respective signal (Figure 10b,c). The maximum intensity and response of cells was observed in the middle of each area. The control area (Figure 10d) had only very little signal from either hypoxia or nitric oxide, as expected.

## 4. Conclusions

In this study, we generated a hypoxic microenvironment and delivered nitric oxide simultaneously to the cells by pumping oxygen scavenger solution and SNP in interdigitated channels. The hypoxia and nitric oxide start to increase after 100 min pumping in the single channel. Both signals remained elevated for 5 h until the end of the experiments. Simultaneous micro patterning of both the hypoxia and the nitric oxide microenvironment in the ≈100 µm size scale defined by the geometry of the channels and the gaps between the channels was demonstrated. Of the two gap dimensions used, 150 µm and 650 µm, the treatments fully covered the former, but not the latter. This shows that, by tailoring the dimensions of the gaps, two types of microenvironments can be created: areas with uniform treatment coverage and areas where the cells are exposed to higher and lesser doses of the treatment.

The advantage of the two-channel system is that cells are not exposed to the scavenger/donor chemicals and that both treatments can be patterned separately, so possible chemical reactions between the donor/sink chemicals are eliminated. The simultaneous manipulation of more than one cellular gaseous microenvironment by a microfluidic chip can be useful for studies in which there is need for precise controlling of hypoxia and nitric oxide in the cells, such as in the early stages of wound healing. In addition to channel geometry-based patterning demonstrated in this study, there is also the possibility to further define the microenvironments by exploiting the fact that the NO delivery is light activated. In this study, we always exposed the whole cell reservoir to light, but it would be possible to also utilize a mask or another method to choose which areas of the channels are exposed to light, thus further fine-tuning the nitric oxide microenvironment.

## Supplementary Materials

The following are available online at https://www.mdpi.com/2072-666X/11/11/979/s1. Supplementary Video S1: Oxygen scavenger solution pumped for 2 h in the one channel. After observing hypoxia signal in the cells, SNP pumped in the other channel. The dual (NO-hypoxia) response in the cells started after 1 h simultaneous pumping of oxygen scavenger solution and SNP. Fluorescence signal from hypoxia and nitric oxide in the cells remains for 5 h.
